# Parents' anti‐Black messages, empathic reactions toward racism, fear of Black individuals, and perceived ability to engage in anti‐racism advocacy among Asian American emerging adults

**DOI:** 10.1111/famp.13062

**Published:** 2024-09-27

**Authors:** Brian TaeHyuk Keum, Lianne Jean Wong, Emma Tran, Mary Minh Giao Nguyen, Cathy Zhu

**Affiliations:** ^1^ Department of Counseling, Developmental & Educational Psychology Boston College Chestnut Hill Massachusetts USA; ^2^ Department of Community Health Sciences University of California Los Angeles California USA; ^3^ Department of Social Welfare University of California Los Angeles California USA; ^4^ Department of Public Policy University of California Los Angeles California USA

**Keywords:** advocacy, anti‐Black racism, anti‐Blackness, anti‐racism, Asian Americans, empathy, parents

## Abstract

Asian Americans, historically oppressed and influenced by White supremacist norms, may internalize anti‐Blackness (beliefs of behaviors that minimize, marginalize, or devalue Black individuals) as they navigate White‐dominated environments to survive and seek acceptance. However, there is limited research addressing the intergenerational socialization of anti‐Blackness within Asian American communities and its impact as a barrier to cross‐racial solidarity and involvement in anti‐racism efforts. Thus, we tested whether parents' anti‐Black messages were associated with fear of Black individuals and lack of empathic reactions to anti‐Black racism, and in turn, related to hindrance in the perceived ability to engage in anti‐racism advocacy among Asian American emerging adults. With data from 205 participants (*M*age = 19.92, *SD* = 2.64, online convenience sample), we conducted a path analysis of parents' anti‐Black messages indirectly associated with perceived ability in advocacy against anti‐Black racism through fear of Black individuals and empathic reactions to anti‐Black racism. Parents' anti‐Black messages were associated with greater fear of Black individuals, which was associated with lower empathic reactions to racism, and in turn, ultimately associated with a lower perceived ability to engage in advocacy against anti‐Black racism. This pathway was the best‐fitting model compared with an alternative parallel model (fear and empathy as separate mediators) and a model with empathy as the first mediator. Our study suggests that clinicians, educators, and researchers should target parents' anti‐Black messages and Asian American emerging adults' emotional responses (fear, empathy) to anti‐Black racism in disrupting anti‐Blackness at parental/family and individual levels.

Anti‐Blackness—beliefs of behaviors that minimize, marginalize, or devalue Black individuals–is deeply embedded in the fabric of the United States (US) society; it is present among White individuals and racially and ethnically minoritized groups, including Asian American individuals (Matriano et al., 2021; Wang & Santos, 2023). The presence of anti‐Blackness in Asian American communities may be explained by the racial triangulation theory, which illustrates the intersectional positionality of White, Asian Americans, and Black individuals in the US (Kim, 1999; Wang & Santos, 2023). According to this theory, White individuals, positioned at the top of the racial hierarchy, valorize Asian Americans as the “model minority” prevailing over Black individuals who are inferior and positioned at the bottom of this hierarchy (Kim, 1999). At the same time, White individuals regard Asian Americans as “unassimilable foreigners,” positioning them beneath White individuals, while Black individuals are considered assimilable and accepted as insiders (Kim, 1999). Thus, racial tensions persist between Asian American and Black communities as they are situated in opposition to each other (Kim, 1999; Wang & Santos, 2023). Some Asian Americans have also been represented by divisive labels such as “honorary Whites” and are often pitted against the legitimacy and struggles of other racially and ethnically minoritized groups such as Black individuals in the US (Wang & Santos, 2023).

It is through these various mechanisms of racial positioning, internalized stereotypes, and socialization in White‐dominated spaces that pressure some Asian Americans to develop anti‐Blackness (Yi & Todd, 2021). Moreover, these stereotypes are partly the result of a White supremacist society taking advantage of a small group of ultra‐successful Asian Americans. The notion of White supremacy serves to defend the “post‐racial” societal narrative (i.e., the model minority myth) that racially and ethnically minoritized groups can attain unmitigated success if they work hard and that structural racial inequities favoring White individuals are minimal (Yellow Horse et al., 2021).

While we must acknowledge and dismantle the role of White supremacy in this conundrum at the systemic level, we call attention to the need to identify, reflect, and challenge anti‐Blackness at the individual and community level (Seider et al., 2020). In doing so, we can work toward dismantling anti‐Blackness and disrupting the influences of White supremacist systems among Asian American communities, despite the discomfort and tension it brings. Given the urgency of fostering critical consciousness, it is essential that we explore how anti‐Blackness is socialized within Asian American communities to understand the process in which Asian American individuals reflect on their own beliefs, attitudes, and behaviors toward Black individuals and communities. However, there is very little research on how anti‐Blackness is socialized within Asian American communities and how it can function as a barrier to cross‐racial solidarity and engagement in anti‐racism advocacy (Jagpal & Schumacher, 2022).

A critical system of influence is the parents. Recent qualitative studies on the socialization of Asian American individuals (Keum et al., 2023) have shown that parents may promote mistrust and fear of Black individuals to their children based on their ideologies of internalizing Whiteness, whether in the US or due to the colonial roots in their country of heritage prior to immigration. It is likely that Asian Americans who receive consistent messages to avoid and distrust Black individuals develop anti‐Black attitudes that can disrupt their participation in anti‐racism advocacy. We can understand the nuances of this socialization process further through the intersectional lens of the racial socialization framework (Thomas & King, 2007), which scholars have developed to analyze how individuals learn about and form their racial positions based on their parental socialization (e.g., messages, behaviors) experiences (Keum et al., 2023). This framework acknowledges the intersection and interaction of salient identities (e.g., race and gender), recognizing that individuals' experiences and identity formation are complex, as their identities can both converge or overlap while also influencing each other to form an individual's unique racialized experiences. Thus, to examine this process among Asian American emerging adults, we tested whether parents' anti‐Black messages were associated with fear of Black individuals and lack of empathic reactions to anti‐Black racism, and in turn, related to hindrance in the perceived ability to engage in anti‐racism advocacy.

## Anti‐Blackness among Asian Americans

Anti‐Blackness is entrenched in Western society and continues to be transmitted from generation to generation and buttressed through the internalization of cultural inferiority (Juang et al., 2017; Keum et al., 2023). Anti‐Blackness is rooted in a long history of Western colonialism, imperialism, capitalism, and militarism in the US (Juang et al., 2017). It is through these hegemonic ideologies and practices that a colonial mentality—and thus, anti‐Blackness—operates and is perpetuated among Asian American communities, justifying White supremacy (Juang et al., 2017; Matriano et al., 2021). Tracing the transmission of anti‐Blackness between generations of Asian American families allows us to better understand the emergence of fear of Black people, and on a systemic scale, fear of Blackness. Anti‐Black stereotypes are constructed as a lack of hard work, laziness, anger, danger, and ingratitude (Purdie‐Vaughns & Williams, 2015). As Fields and Fields (2012) wrote, These racist tropes of a Black criminal subclass are now so ingrained in the fabric of. American society that science long ago confirmed the existence of a pervasive, unconscious, and largely automatic bias against dark‐skinned individuals as more hostile, criminal, and prone to violence. These biases infect nearly everyone. (p. 934).

These stereotypes live not only to imbue the inferiority of Black communities in a racial hierarchy but also to superimpose onto individual people who are racialized as Black, resulting in stigmatized identities (Brezina & Winder, 2003). Consequently, under the guise that performing the “model minority” will precipitate societal inclusion (which, in turn, begets educational and financial success), and therefore, a protected spot on the racial ladder, Asian American families may pass messaging to their younger generations to reinforce this racial hierarchy through anti‐Black stereotypes and fear‐mongering.

## Parental socialization of anti‐Blackness

Recent research has called attention to parents as a major influence in Asian American youths' and emerging adults' development of anti‐Black attitudes (Juang et al., 2017; Keum et al., 2023), which suggests that parental socialization can function as a vehicle for anti‐Blackness to subsist across generations. Parents are commonly regarded as the primary socialization agent, consciously or unconsciously aiding their children in developing their identities, roles, and responsibilities for adequate functioning in society (Thomas & King, 2007). Through parental socialization, values, beliefs, and ideas that support anti‐Blackness can hamper children's pro‐social behaviors, including engagement in anti‐racism advocacy and cross‐racial solidarity (Thomas & King, 2007). For example, Lee et al. (2022) found anti‐Blackness to be both pervasive among families of Chinese American youth and a barrier to their engagement in anti‐racist political activism. In this study, a 9th grader shared his family's perspectives:

They hold a very strong belief that all African Americans are criminals, they're drug addicts, they're supposed to be in jail. Especially my father. He mentioned it to me once that if I marry an African American woman, he will use a broom to get me out of the house. (p.1075).

Aware of this problematic perspective, the 9th grader continues to share his struggles with changing his father's beliefs, highlighting the complicated conditions that thwart youth's anti‐racist development. Another participant described how her parents discouraged her from political engagement because they viewed it as dangerous and told her to “lay low” (Lee et al., 2022). The parents' request for their daughter to keep a low profile reinforces the model minority stereotype among Asian Americans to remain silent as a defense mechanism for minimizing the harmful impact of racism (Shih et al., 2019), which consequently downplays the struggles Black individuals experience as a result of White dominant oppressive systems (Lee et al., 2022). The same participant also recounted how her parents advised her against talking about racism at home yet encouraged her to speak up about racism only if she herself experienced a racist incident (Lee et al., 2022). The socialization messages from parents, as illustrated in this study, not only illuminate Asian American parents' anti‐Black sentiments but also demonstrate how parents' anti‐Black beliefs can inhibit children's critical consciousness development and engagement in anti‐racist learning and advocacy (Lee et al., 2022).

Studies have documented how anti‐Blackness is socialized within the parent–child relationships of Asian American families. For example, Keum et al. (2023) employed an intersectional approach to qualitatively examine the gendered racial socialization messages Asian American men received growing up and found that participants' parents expressed aversion to dating darker‐skinned women, indicating their parents' internalized racism and acceptance of a racial hierarchy in which people of darker skin color, such as Black individuals, are racially inferior. Similarly, Ahn et al. (2022) found that Asian American women participants were explicitly told by their parents not to date or marry Black men while dating White men was an exception. These examples suggest that Asian American parents' approval of dating White partners may reflect the colonial mentality that favors close proximity to Whiteness and the power and privilege associated with being White by exercising anti‐Black preferences (Ahn et al., 2022; Keum et al., 2023). Thus, the development of anti‐Blackness may be contextualized uniquely within the intersection of racism and sexism Asian American women and men experience across generations (Keum et al., 2022; Keum, Brady, et al., 2018; Keum, Miller, et al., 2018).

## Anti‐Blackness and fear of Black individuals

Racial socialization scholarship has shown that while parents share messages about race and ethnicity intending to promote cultural pride and safety, they also may share race‐related messages that can teach children to be mistrustful and apprehensive about people of other races (Thomas & King, 2007). These processes have important implications for longitudinal, developmental study; Asian American children may inherit both direct and indirect messaging that encourages them to participate in a double‐edged enterprise to remain a model minority and to avoid and fear Black people. This fear has roots in how Black people are taxonomized as what Black studies scholar Katharine McKittrick calls the “absolute Other”–an untouchable class with a contagious pathology that jeopardizes one's position on the racial hierarchy ladder if they show alliance with, empathy for, or proximity to Black people (McKittrick, 2011). A recent experiment, for example, found that Asian parents are more likely to select for their children to attend schools with primarily White racial composition, whereas Latinx and Black respondents preferred for their children to attend Latinx and Black schools, respectively, over White schools (Hailey, 2022). Furthermore, Asian Americans are more likely to live in White neighborhoods than in Black neighborhoods (Iceland et al., 2010). To reiterate, both concepts—of Asianness and Blackness—are constructed to produce and reify the supreme norm that is Whiteness, which itself is constructed. As such, Asian Americans, in pursuit of survival and success in the US, perform not only Whiteness and model minority but also fear and avoidance of Black people.

## Empathic reactions to anti‐Black racism and anti‐racism advocacy

As empathy can promote pro‐social behavior, such as helping, collaboration, understanding, appreciation, and cooperation between people of different groups (Forgiarini et al., 2011), it has become an increasingly important topic of study for researchers and interventionists seeking to reduce intergroup conflict and antipathy. Research has shown that empathy can encourage advocacy on social justice issues, for example, by bolstering support for policies that would benefit the welfare of other groups (Batson & Ahmad, 2009). While definitions of empathy vary, researchers agree that it involves several layers of cognitive and affective processing and can emerge in different conditions and take different forms. As such, research that focuses on barriers to empathy for certain groups in certain environments is important in the pursuit of intergroup harmony and social justice. For Asian Americans, this realm of research is underexplored. Recent research shows that Asian American emerging adults exposed to storytelling on anti‐Black racism may feel pain and sadness to arouse a sense of injustice and compassion (Keum et al., 2024). However, as empathy may be inhibited by color‐blind racial attitudes and fear of or antagonism toward Blackness, these factors may hinder Asian Americans' pursuit of cross‐racial solidarity. Color‐blind racial attitudes operate in tandem with anti‐Black attitudes as the dismissal of racialization, ultimately erasing the reality that White supremacist systems in the US have oppressed Black communities (Keum, Brady, et al., 2018; Keum, Miller, et al., 2018). Messages of anti‐Blackness passed down through Asian American families, then, may be an important interpersonal and intergroup mechanism that may uphold the racial order by conveying Black individuals first as discretely distinct from other groups (i.e., ignoring relational formations of race) and second as out‐group threats, or those to be feared (i.e., stereotyping and ascribing threat and danger to an entire group of people). These messages of out‐group threat may facilitate antipathy toward observed anti‐Black racism, posing a considerable challenge to engagement in advocacy. For instance, Yi et al.'s (2023) meta‐analysis showed that color‐blind racial attitudes were associated with more anti‐Black prejudice, fewer anti‐racist behaviors, and lower racial/ethnocultural empathy. These findings suggest that racial attitudes are related to empathy, which ultimately be an important precursor to engagement with anti‐racist advocacy.

## The present study

Anti‐Blackness is a complex and intergenerational issue among Asian American communities that can act as a barrier toward cross‐racial solidarity and anti‐racism advocacy. Yet, little is known about how anti‐Blackness manifests itself through parental influence on an Asian American individual. Based on emerging scholarship on the socialization experiences of Asian American individuals, we focused on the role parents may have in imparting anti‐Blackness messages toward their children and how these messages may be ultimately associated with hindrance on anti‐racism advocacy among Asian American emerging adults. In understanding this relationship more closely, we examined the fear of Black individuals and empathic reactions to racism as mediators. Identifying mediators in this pathway is crucial as it can help inform future interventions to mitigate the negative implications of parents' anti‐Blackness messages. Specifically, we hypothesized that parents' anti‐Blackness messages would be indirectly associated with lower anti‐racism advocacy (Hypothesis 1) through fear of Black individuals and empathic reactions to racism (Hypothesis 2; Greater parents' anti‐Blackness messages ‐> Greater fear of Black individuals ‐> Lower empathic reactions to racism ‐> Lower anti‐racism advocacy). In analyzing this path model, we also explored an alternative parallel mediation model (separate indirect relations through fear of Black individuals or empathic reactions) to test whether the proposed sequential relationships would indeed be the best‐fitting model.

## METHOD

### Participants

A cross‐sectional convenience sample of 205 Asian American college students (*M*age = 19.92, *SD* = 2.64) provided data. The majority of the sample identified as a woman (67.8%, *n* = 135), while the remainder identified as a man (31.2%, *n* = 62), transfeminine (0.5%, *n* = 1), and other (0.5%, *n* = 1). About 81.5% identified as heterosexual (*n* = 159), 6.7% bisexual (*n* = 13), 4.1% uncertain (*n* = 8), 3.1% gay (*n* = 6), 1.5% asexual (*n* = 3), 1% lesbian (*n* = 2), 1% queer (*n* = 2), 0.5% pansexual (*n* = 1), and 0.5% other (*n* = 1). The sample was diverse in ethnicity and consisted of 31.8% Chinese (*n* = 63), 11.6% multiracial and/or multiethnic (*n* = 23), 11.1% Indian (*n* = 22), 10.1% Filipino (*n* = 20), 10.1% Vietnamese (*n* = 20), 6.6% Japanese (*n* = 13), 6.6% Korean (*n* = 13), 3.5% Taiwanese (*n* = 7), 3% other (*n* = 6), 2% Native Hawaiian or Pacific Islander (*n* = 4), 1% Singaporean (*n* = 2), 1% Cambodian (*n* = 2), 1% Indonesian (*n* = 2), and 0.5% Bangladeshi (*n* = 1). About 50.5% (*n* = 100) identified as second generation (i.e., born in the US and at least one parent is an immigrant), 24.7% (*n* = 49) first generation (i.e., born outside of US), 11.1% (*n* = 22) third generation, 5.6% (*n* = 11) 1.25 generation (i.e., immigrated between 13 and 17 years of age), 3.5% (*n* = 7) 1.75 generation (i.e., immigrated between 0 and 5 years of age), 2.0% (*n* = 4) 1.5 generation (i.e., immigrated between 6 and 12 years of age), 1.5% (*n* = 3) adoptee, and 1.0% (*n* = 2) other. About 66.3% (*n* = 132) of participants' parents immigrated to the US while 33.7% (*n* = 67) were born in the US. About 38.3% (*n* = 74) of the participants identified as upper middle class, 34.2% (*n* = 66) as middle class, 12.4% (*n* = 24) as working class, 9.8% (*n* = 19) as lower class, 4.7% (*n* = 9) as upper class, and 0.5% (*n* = 1) as other.

### Procedure

The study was approved by the Institutional Review Board at the University of California, Los Angeles. Data were collected between December 2020 and December 2021. Participants were recruited via the SONA subject pool at a public university on the West Coast and a public university in the Mid‐Atlantic Region. The inclusion criteria were: (a) 18 years old or older, (b) self‐identify as an Asian American, and (c) reside in the US. Data were collected via an online survey consisting of informed consent, study variable measures, and demographic items hosted by Qualtrics. The survey was advertised as an examination of racial issues among Asian Americans through university listservs and the SONA subject pool. Those who participated through SONA received course credit for their participation.

### Measures

#### Parents' anti‐Black messages

We adapted the Promotion of Mistrust subscale (three items) from the Ethnic‐Racial Socialization Scale (Hughes & Johnson, 2001; Tran & Lee, 2010) to measure anti‐Black messages participants received from their parents. The three items were adapted as follows: “Told you to avoid Black people,” “Done or said things to encourage you to keep a distance from Black people,” and “Done or said things to you to keep you from trusting Black people.” Following the measure's logic, we also added two items to expand the breadth of the anti‐Black messages based on the literature: “Told you that Black people are dangerous,” “Told you that you can't be friends with Black people.” Items were rated on a 5‐point Likert‐type scale ranging from 1 (*never*) to 5 (*always*). Items were summed, with higher scores indicating greater anti‐Black messages received from the parents. The Cronbach's alpha for the current sample was 0.92.

#### Fear of black individuals

We adapted three items from the White Fear of Others subscale from the Psychosocial Costs of Racism to Whites scale (PCRW; Spanierman & Heppner, 2004) to measure participants' fearful attitudes toward Black individuals. The items are: “I often find myself fearful of Black people,” “I am distrustful of Black people,” and “I have very few friends who are Black.” Items were rated on a 7‐point Likert‐type scale ranging from 1 (*strongly disagree*) to 7 (*strongly agree*). Items were summed, with higher scores indicating greater fear toward Black individuals. The Cronbach's alpha for the current sample was 0.72.

#### Empathic reactions toward racism

We adapted the White Empathic Reactions toward Racism subscale from the Psychosocial Costs of Racism to Whites scale (PCRW; Spanierman & Heppner, 2004) to measure participants' ability to critically recognize the issue of racism and have empathy regarding racism and racial injustice. All six items were administered without any changes except item five, which was removed as it did not fit the context of our participants: that is, “Racism is dehumanizing to people of all races, including Whites.” Sample items include: “I am angry that racism exists,” “It disturbs me when people express racist views.” Items were rated on a 7‐point Likert‐type scale ranging from 1 (*strongly disagree*) to 7 (*strongly agree*). Items were summed, with higher scores indicating greater empathic reactions toward racism. The Cronbach's alpha for the current sample was 0.86.

#### Perceived ability in advocacy against anti‐Black racism

The 5‐item Perceived Behavioral Control (PBC) subscale of the Social Justice Scale (Torres‐Haring et al., 2012) was adapted to measure participants' perceived ability to perform and engage in advocacy against anti‐Black racism. We modified the items so that the items' reference was toward Black individuals (e.g., “Others” changed to “Black individuals”). Sample items include: “I am certain that I possess an ability to work with Black individuals and groups in ways that are empowering,” “I feel confident in my ability to talk to others about social injustices and the impact of social conditions on Black individuals' health and well‐being.” Items were rated on a 7‐point Likert‐type scale ranging from 1 (*strongly disagree*) to 7 (*strongly agree*). Items were summed, with higher scores indicating a greater perceived ability to engage in advocacy against anti‐Black racism. The PBC scores have been shown to significantly predict people's intentions to engage in social justice advocacy (Torres‐Haring et al., 2012). The Cronbach's alpha for the current sample was 0.86.

### Data analysis

A total of 357 individuals participated in the survey. Of these, 47 were removed for not providing consent, 14 were removed for not providing any data, and 24 were removed for not identifying as Asian. Little's MCAR test was not significant, *χ* ^2^ = 718.46, *df* = 665, *p* = 0.074, suggesting that data were missing completely at random. Of the remaining cases, 67 were removed for missing more than 20% of the data. The final sample size was 205 (57% of total responses), with just two cases missing 16% of the data. Little's MCAR test was not significant, *χ* ^2^ = 103.303, *df* = 117, *p* = 0.813, suggesting that data were missing completely at random. Mardia's multivariate skewness and kurtosis test (skewness = 112.49, *z* = 3843.39, *p* < 0.001; kurtosis = 519.62, *z* = 30.57, *p* < 0.001) suggested nonconformity to the multivariate normality assumption (Cain et al., 2017). Thus, we used maximum likelihood estimation with robust standard errors in our analyses. We handled missing data with full‐information maximum likelihood in Mplus (Enders, 2010).

We conducted a Monte Carlo simulation analysis (Schoemann et al., 2017) to examine whether our sample size would provide adequate statistical power. We drew effect size estimates for each of the proposed paths (ranging from 0.10 to 0.50 as small to moderate effect sizes were previously reported) from previous empirical studies (e.g., Keum et al., 2022; Pieterse et al., 2016). Results after 1000 repetitions indicated that a sample size of 205 would yield a desired power of 0.80. In terms of estimation accuracy, all estimation bias rates were below the recommended 0.05 threshold. The 95% coverage rates were also above the recommended 0.90 threshold, which supported the stability and accuracy of the yielded findings for the overall model.

**FIGURE 1:**
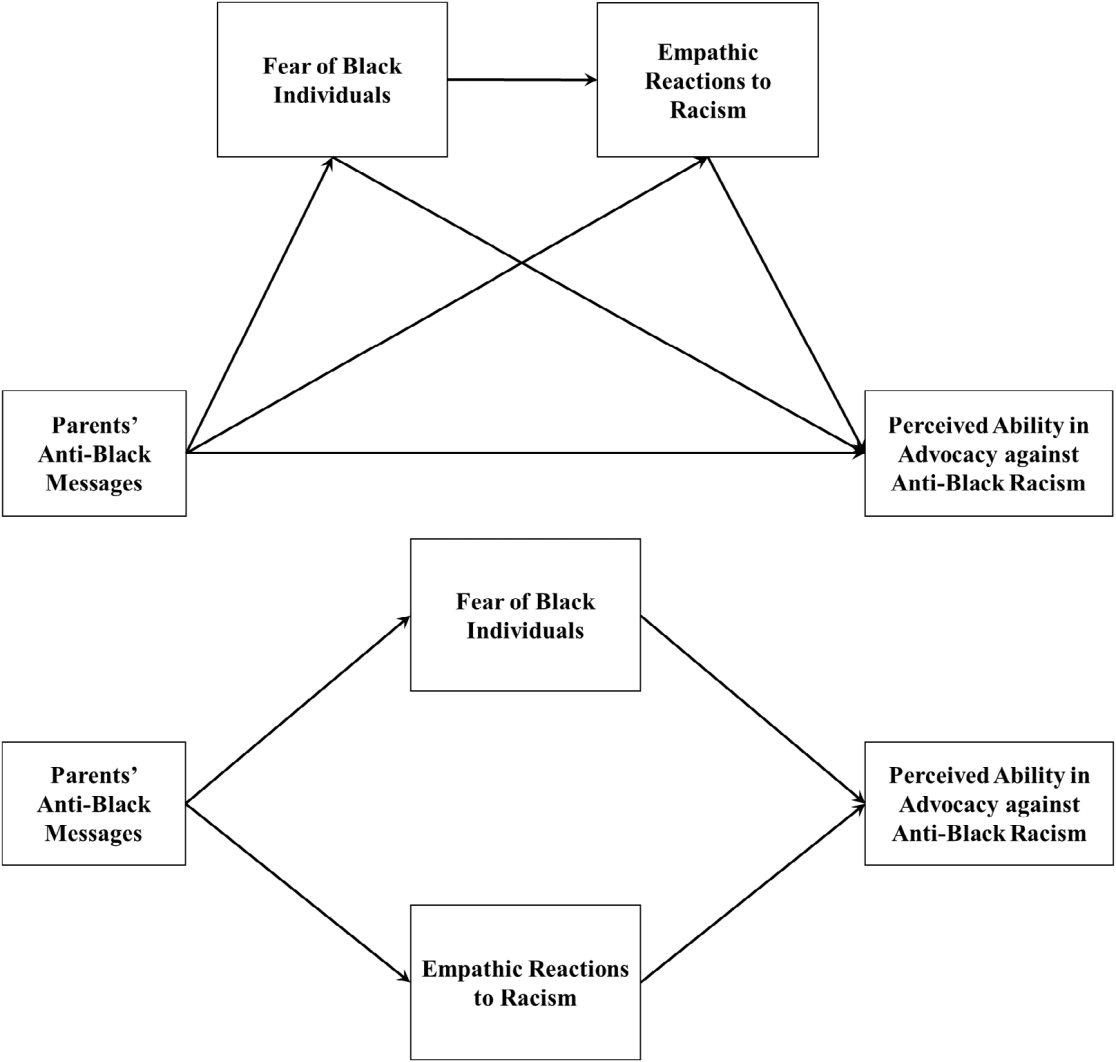
Hypothesized serial model and alternative parallel model. Bottom, alternative parallel model; Top, hypothesized serial model.

We tested our hypothesized serial mediation model (Figure 1) using the latent variable path analysis in M*plus* 8.7 (Muthen & Muthen, 2017). We specified Parents' anti‐Black messages (PABM) as the independent variable, Fear of Black Individuals (FBI) and Empathic Reactions toward Racism (ERR) as serial mediators (FBI‐>ERR), and Perceived ability in advocacy against anti‐Black racism (PAAABR) as the dependent variable. We controlled for generational status, parents' immigration status, and socioeconomic status. We evaluated the model fit using the Yuan–Bentler (YB) scaled *χ* ^*2*^‐test and several approximate fit indices (Hu & Bentler, 1999): (a) the root mean square error of approximation (RMSEA; close to <0.08 for “acceptable” fit); (b) the comparative fit index and Tucker–Lewis fit index (CFI, TLI; close to 0.95 for “good” fit); and (c) the standardized root mean square residual (SRMR; close to <0.08 for “acceptable” fit). To examine specific path coefficients and indirect (i.e., mediation) effects, we followed best practices (Hayes & Scharkow, 2013) and adopted the bootstrap method using 5000 random samples. We used 99% confidence intervals (CI) to assess the statistical significance of the mediation effects where CIs excluding 0 were deemed equivalent to *p* < 0.01. To rule out the alternative parallel pathway (Figure 1), we ran the model with ERR and FBI as parallel mediators and assessed the model fit against the hypothesized serial model using the Yuan–Bentler (YB) scaled *χ* ^*2*^‐test and comparing the Akaike and Bayesian Information Criterion (AIC, BIC) values. Additionally, to assess and rule out the alternative serial mediator sequence, we also ran the serial model with the order of the mediators flipped (ERR‐>FBI).

## RESULTS

Table 1 contains bivariate correlations, internal reliability estimates, and descriptive statistics. Cronbach's alphas for all measures were adequate. PABM correlated positively with FBI with a moderate to large effect and negatively with PAAABR with a small effect. FBI was correlated negatively with ERR with a small effect and PAAABR with a moderate to large effect. ERR was correlated positively with PAAABR with a moderate effect.

**TABLE 1:** Descriptive statistics and bivariate correlations of study variables.

Variables	Min	Descriptives and correlations						3
		Max	*M*	*SD*	*α*	1	2	
1. PABM	5.00	25.00	9.60	4.56	0.92			
2. FBI	3.00	21.00	8.53	3.87	0.72	0.42**		
3. ERR	5.00	35.00	30.57	4.44	0.86	−0.12	−0.20**	
4. PAAABR	5.00	35.00	26.48	4.63	0.86	−0.21**	−0.40**	0.32**

Abbreviations: ERR, Empathic Reactions toward Racism; FBI, Fear of Black Individuals; PAAABR, Perceived Ability in Advocacy against Anti‐Black Racism; PABM, Parents' Anti‐Black Messages.

**p < 0.01.

### Latent variable path analysis

Before testing our hypothesized path models, we assessed the measurement model of all our study variables in their latent variable specifications by conducting confirmatory factor analysis. All variables were specified to be unidimensional. The measurement model yielded a good fit, RMSEA = 0.045 [0.029, 0.058]; CFI = 0.97; TLI = 0.96, SRMR = 0.049, suggesting that the adapted measures fit the data well. We proceeded to test the fit of the hypothesized serial model (PABM‐>FBI‐>ERR‐>PBC). The serial model fits the data well: $\chi_{\mathrm{YB}}^{2}$= 299.71, *df* = 200, *p* < 0.001; RMSEA = 0.051 [0.039, 0.063]; CFI = 0.95; TLI = 0.94; SRMR = 0.059. The alternative parallel model fit the data adequately: $\chi_{\mathrm{YB}}^{2}$= 318.68, *df* = 201, *p* < 0.001; RMSEA = 0.055 [0.043, 0.066]; CFI = 0.94; TLI = 0.93; SRMR = 0.075. The $\chi_{\mathrm{YB}}^{2}$ difference test suggested that the serial model fit the data significantly better than the parallel model, Δ*χ* ^2^ = 15.13, Δ*df* = 1, *p* < 0.001. The AIC and BIC values for the serial model (9141.41, 9147.34) were lower than the parallel model (9160.04, 9165, 88) by more than 10 units, suggesting that the serial model fit the data better than the parallel (Burnham & Anderson, 2004). Thus, we retained our hypothesized serial model for further testing and ruled out the alternative parallel model.

**FIGURE 2:**
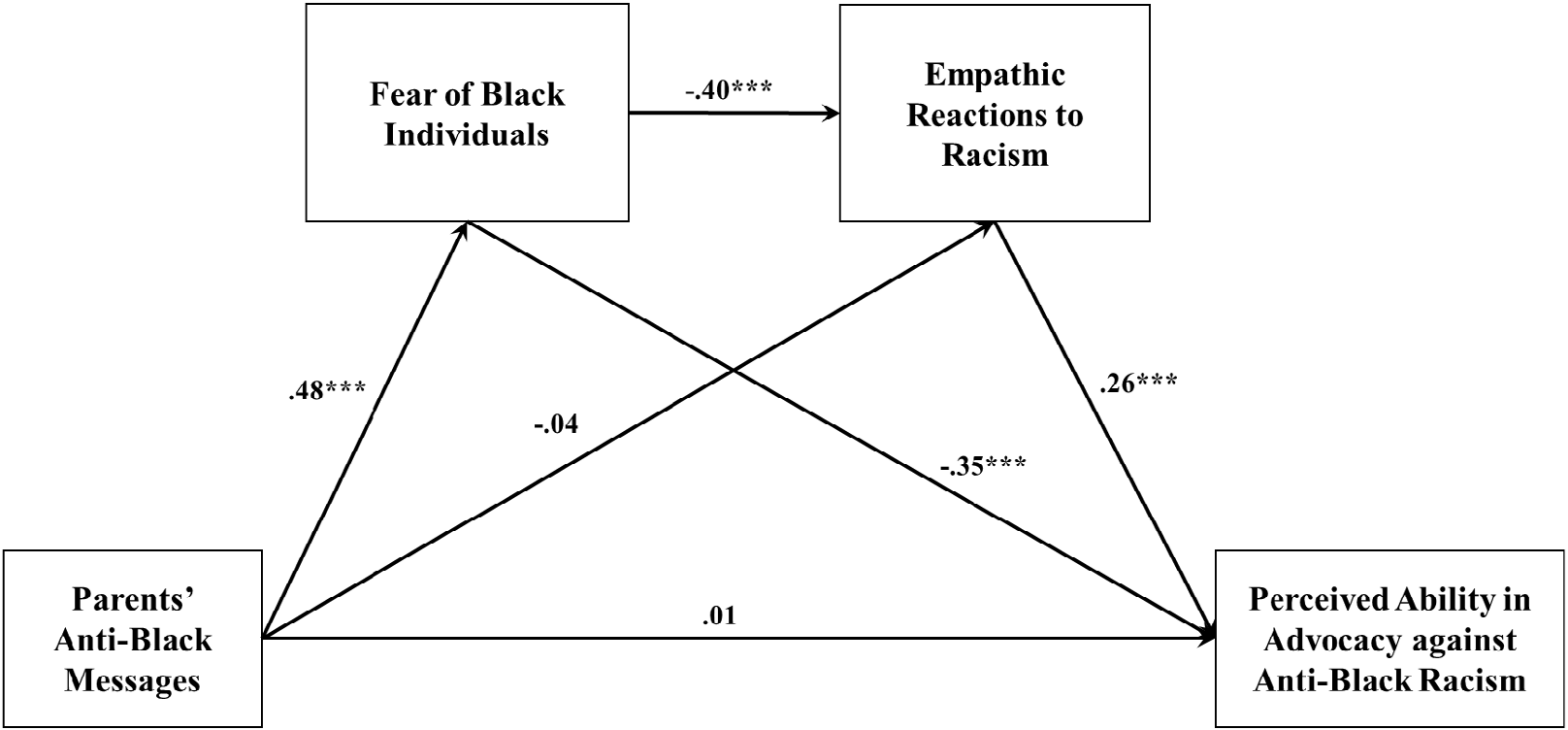
Path estimates for the hypothesized serial model. ***p < 0.001.

Figure 2 lists the completely standardized path coefficients, and Table 2 lists the total direct, total indirect, and specific indirect effects. PABM significantly associated with PAAABR (standardized effect *β* = −0.221, 99% bootstrapped CI = [−0.437, −0.005]). The total effect was decomposed into a nonsignificant direct effect (*β* = 0.008, 99% bootstrapped CI = [−0.203, 0.220]) and a significant total indirect effect through the hypothesized mediators (standardized total indirect effect *β* = −0.229, 99% bootstrapped CI = [−0.387, −0.071]) that explained 51% of the total effect. The indirect pathway from PABM to ERR via FBI was significant (standardized total indirect effect *β* = −0.193, 99% bootstrapped CI = [−0.326, −0.061]). The indirect pathway from FBI to PAAABR via ERR was significant (standardized total indirect effect *β* = −0.106, 99% bootstrapped CI = [−0.201, −0.010]). Finally, the hypothesized full indirect pathway PABM‐>FBI‐>ERR‐>PAAABR was significant (standardized total indirect effect *β* = −0.051, 99% bootstrapped CI = [−0.100, −0.001]). When we ran the serial model with the order of the mediators flipped (ERR‐ > FBI), the indirect effects were not significant (Table 2), which made sense given that this order was not in line with our conceptualization and hypothesis. These results supported our hypothesis that greater PABM was ultimately associated with lower PAAABR through greater FBI and lower ERR. The significance of the mediators was substantiated, given the nonsignificant direct effect. The model accounted for 27% of the variance in PAAABR.

**TABLE 2:** Estimate of indirect effects from bootstrap analysis.

IV	Mediator(s)	DV	Standardized effect estimate	*SE*	99% bootstrap CI
PABM → FBI → ERR → PAAABR					
Total Effect					
PABM		PAAABR	−0.221**	0.08	[−0.437, −0.005]
Total Direct Effect					
PABM		PAAABR	0.008	0.08	[−0.203, 0.220]
Total Indirect Effect					
PABM		PAAABR	−0.229**	0.06	[−0.387, −0.071]
Specific Indirect Effect					
PABM	→ FBI →	ERR	−0.193**	0.05	[−0.326, −0.061]
FBI	→ ERR →	PAAABR	−0.106**	0.04	[−0.201, −0.010]
PABM	→ FBI → ERR →	PAAABR	−0.051**	0.02	[−0.100, −0.001]
PABM → ERR → FBI → PAAABR					
Total Effect					
PABM		PAAABR	−0.221**	0.08	[−0.437, −0.005]
Total Direct Effect					
PABM		PAAABR	0.008	0.08	[−0.203, 0.220]
Total Indirect Effect					
PABM		PAAABR	−0.229**	0.06	[−0.387, −0.071]
Specific Indirect Effect					
PABM	→ ERR →	FBI	0.075	0.05	[−0.026, 0.175]
ERR	→ FBI →	PAAABR	0.116**	0.04	[0.007, 0.225]
PABM	→ ERR → FBI →	PAAABR	−0.026	0.02	[−0.067, 0.015]

Abbreviations: ERR, Empathic Reactions toward Racism; FBI, Fear of Black Individuals; PAAABR, Perceived Ability in Advocacy against Anti‐Black Racism; PABM, Parents' Anti‐Black Messages.

**p < 0.01.

## DISCUSSION

The current study examined whether parental transmission of anti‐Black messages would hinder Asian American emerging adults' perceived ability to engage in advocacy against anti‐Black racism. As hypothesized, we found that PABM were associated with greater FBI, which was associated with lower empathic reactions to racism, and in turn, associated with a lower perceived ability to engage in advocacy against anti‐Black racism. This pathway was the best‐fitting model compared with an alternative parallel model (fear and empathy as separate mediators) and a model with empathy as the first mediator. The findings underscore how anti‐Blackness could be socialized among Asian American emerging adults by their parents and emphasize the need to develop strategies to disrupt anti‐Blackness at parental and family levels. The resulting FBI and lack of empathic reactions to racism could serve as intervention points for helping Asian American emerging adults develop their anti‐racist identity.

As the first part of the path model shows, Asian American emerging adults who received greater anti‐Black messages from their parents expressed greater fear toward Black individuals. This fear may take the form of avoidance or discomfort in interactions with or in racially charged conversations related to Black individuals. This may be explained by the internalization of the model minority myth, which “fosters interminority intergroup competition to maintain the existing racial hierarchy” (Lei et al., 2022, p. 3) and reinforces the racial divide between Asian American and Black communities. Given the historical pervasiveness of anti‐Blackness and the ongoing portrayal of Black communities in the media through negative stereotypes (e.g., criminals, gang members), Black individuals are often perceived as threats. According to Stephan and Finlay (1999), prejudice and stereotypes result in exaggerated perceptions of difference, which is often associated with heightened levels of fear and threat toward outgroups (i.e., Black individuals in this context).

The FBI may also have a negative downstream impact on Asian American emerging adults' ability to have empathy toward anti‐Black racism and engagement in anti‐racism advocacy. Research suggests (Keum et al., 2024) that Asian American emerging adults may develop greater compassion and understanding toward Black communities experiencing racism and racial violence through empathy. Empathy increases one's ability to understand others' experiences through a more compassionate lens or perspective taking (Forgiarini et al., 2011). Although there may not be a complete understanding of each other's experiences of racism, recognizing the shared experiences can be used as a collective resistance against racism (Matriano et al., 2021). Furthermore, empathy may serve as a key emotion in helping Asian American emerging adults develop a foundation to engage in cross‐racial solidarity and partnerships to dismantle systemic racism and White supremacy (Thomas & King, 2007). However, as we found, PABM and the resulting FBI may hinder the development of empathy among Asian Americans, limiting the potential to drive engagement in anti‐racism advocacy.

Our study demonstrates that fear is an important emotional response to consider in interracial interactions, perhaps both real and imagined, between Asians and Black individuals. Given the nature of the items in our construct of fear, there is a range of meanings as to what this fear represents. For example, it may be possible that respondents harbor fear directly toward Black individuals resulting from their risk assessment of criminal victimization, derived from previous experiences, knowledge, attitudes, and social relations (Ferraro, 1995). Fear could also point to evidence for group threat theories of racial hostility, in which Asians may feel threatened in their superior group position by their perceived inferior position of Black individuals (Blumer, 1958). In either case, our findings signal the importance of fear in explaining antipathy toward anti‐Blackness and corroborate with other study results that show similar patterns (Lee & Ulmer, 2000).

It is important to note that FBI, or of Blackness, is not a homogenous experience but contextually situated in the histories and spatial arrangements of a time and place. Critical geographer Laura Pulido's groundbreaking study of multiracial activists shows that their philosophies about social justice originated in the places where they grew up; the messaging they received from their families, friends, and neighbors depended on who lived nearby. For instance, one young Japanese activist remembered that her mother admonished her, “Don't play with the *kurocha'* (a pejorative for Black children),” for “fear that her Asian daughter would incur even more racism and discrimination if she associated with Blacks” (Pulido, 2006, pp. 57–58). Though our study shows that parental messaging of fear can influence anti‐Black attitudes and practices among their children, children's awareness of this phenomenon may encourage them to seek alternative explanations for anti‐Blackness, such that visions for multiracial solidarity may replace fear.

### Limitations

One main limitation of the current study is using cross‐sectional data which limits us from any consideration of causality or temporal flow of the variables. Relatedly, participants recalled messages from their parents in the past retroactively. There may be some lapse in their recollection of the messages passed down, or participants may not report all the messages due to fear of judgment. Therefore, the anti‐Blackness messages passed down from generation to generation may differ from one family to the other and how the messages manifest themselves into microaggressive or discriminating behaviors. We do not have the parents' perspective nor the initial beginnings of anti‐Blackness messages within each family. Anti‐Blackness messages might differ across different families and generations. Individuals who lack exposure or conversation about racial justice issues pertaining to Black communities, in contrast to individuals who grew up with explicit or direct messages of anti‐Blackness, may differ in experiences. Related to messaging, our measure of anti‐Black socialization focused on explicit verbal messages and does not account for non‐verbal or implicit socialization (e.g., parents' behaviors). Future studies would need to involve verbal and non‐verbal data from parents on how they engage with race‐related issues in their families.

Another limitation of our study is the sample‐related generalizability limitation, as the sample had more representation of the East Asian and heterosexual population. Thus, the findings may be less generalizable to non‐East and LGBTQ+ Asian Americans. For example, South/Southeast Asians' experience with racism may be more contextualized by colorism, low SES, and educational status stereotypes, which could differentiate their perspective on anti‐Black racism compared with East Asians (Yamashita, 2022). Another limitation is the geographical locations (e.g., liberal vs. non‐liberal/progressive states) where anti‐racism advocacy is more prevalent in some communities than others. Depending on the geographic location of the respondents and their upbringing, messages of anti‐Blackness and fear may look different across each population within different regions. Last, in our study, the average age of our sample size was 19 years old. They were also college students who may have been more attuned to the anti‐Black racism issues (e.g., mean empathy score = 30.57; range = 5–35) through educational opportunities and anti‐racist initiatives on campus. Those who are older may experience generational differences in their view on issues of race and racism. In relation, social desirability may have affected participants' reports of explicit attitudes such as FBI. Future studies need to objectively examine the impact of anti‐Black messages received from parents across these demographic factors and whether our findings can be generalizable beyond the major identities in our current sample.

### Implications for future research

While this study makes a much‐needed contribution to the literature on how anti‐Blackness is socialized among Asian American emerging adults by their parents, research remains limited, and further investigation is needed to explore this topic and deepen our understanding of how parental socialization of anti‐Blackness impedes cross‐racial solidarity and engagement in anti‐racism advocacy. To the best of our knowledge, our study is among the first to examine how Asian American emerging adults received anti‐Black messages from their parents. To advance this scholarship, qualitative studies should conduct in‐depth interviews or focus groups with both Asian American emerging adults and their parents to gain a comprehensive understanding of their experiences with, and beliefs and attitudes about, racism and anti‐Blackness. Interview questions should center on learning about the barriers that hinder open discussion and critical reflection about anti‐Blackness and racism. Qualitative studies should also utilize a longitudinal design to examine how anti‐Blackness is socialized at multiple timepoints, allowing researchers to explore other influences in addition to parental socialization, such as peer interactions and exposure to anti‐racist education. Furthermore, to increase the generalizability of this research, comparative studies should examine how anti‐Blackness is socialized across different Asian ethnicities and relevant demographic factors such as generational status.

As our study suggests, increasing engagement in anti‐racism advocacy among Asian American emerging adults in support of Black communities warrants efforts to reduce stereotypical beliefs that instill fearful images of Black individuals and promote empathic reactions to racism. Future research should explore the malleability of the roots of these emotions. For example, studies could identify specific triggers of fear toward Black individuals, including the intensity and frequency of these triggers. The degree of empathy and emotional responses to anti‐Black racism should also be measured. These measurements could be conducted in an observational study where researchers observe participants' reactions and behaviors during racially charged situations or interactions. Collecting these objective measures would help reduce bias from self‐report surveys, and thus, conducting a mixed methods study to collect qualitative and quantitative data would help advance this research.

### Implications for practice and advocacy

In one sense, anti‐Blackness is inextricably linked with Asian Americans' internalized racism; thus, ceasing anti‐Blackness will require interventions that promote critical racial consciousness among Asian American individuals and their families. To cultivate critical consciousness, Chopra (2021) outlines that the following is involved in this process: “(a) becoming aware of messages that have been internalized (e.g., colorism, model minority myth, White supremacy, anti‐Black racism) and the sociopolitical context of oppression, and (b) taking steps individually and/or collectively to combat oppression.”

Interventions that disrupt anti‐Blackness should occur at multiple levels of influence, including at the individual, family, and societal levels. At the individual level, interventions should prioritize anti‐racist education through workshops and training to increase peoples' awareness of anti‐Blackness and how these views can be internalized and dismantled. Incorporating anti‐racist pedagogy into school curricula, especially in primary and secondary schools, is critical for countering the influences of anti‐Black biases in society among youth. In addition to teaching Asian American and Black history, curricula should highlight the history of racial triangulation and Asian‐Black solidarity movements (e.g., the activism of Yuri Kochiyama and Grace Lee Boggs). Furthermore, schools are an ideal place of intervention to foster an inclusive and diverse learning environment in which empathy toward other marginalized racial and ethnic groups could be developed. In turn, this could empower youth toward civic engagement, including participation in anti‐racism advocacy.

In today's digital era, social media content such as digital storytelling approaches could be leveraged to supplement anti‐racist teachings. In a study that employed digital storytelling to foster Asian American emerging adults' advocacy against anti‐Black racism, Keum et al. (2024) found that exposure to emotionally stimulating digital media stories on anti‐Black racism led to a reduction in fear against Black individuals and an increase in empathic reactions to anti‐Black racism, which was associated with greater anti‐racism advocacy intentions. Thus, future interventions could incorporate the platform of storytelling to understand the shared and unique experiences of racism to build collective solidary.

Furthermore, as seen on multiple social media platforms and news outlets across the nation, the COVID‐19 pandemic has led to an alarming rise in hate crimes against Asians, including cross‐racial violence (Wang & Santos, 2023). Asian American communities have harbored acute feelings of fear, hindering their engagement in cross‐racial solidarity. To combat this fear, Asian American emerging adults should “call out” their family members who may engage in harmful conversations toward Black communities if it is safe to do so. Intervention at the family level is crucial for dismantling intergenerational socialization of anti‐Blackness, especially when nonjudgmental spaces are available for facilitating meaningful discourse on critical racial consciousness. These conversations may involve challenging both explicit and implicit racial biases against Black communities by, for example, asking open‐ended questions that require the individual to reflect on their responses and learn to understand where their racially‐motivated comment may have originated (Keum et al., 2023).

For children of Asian American parents, Lei et al. (2022) suggest a “collective racial socialization” approach—a new dimension of ethnic‐racial socialization that involves parents engaging in discussion with their children about finding commonalities in their own discriminatory experiences with those from other racially minoritized groups. This type of parental socialization focuses more broadly on how, despite differences in discriminatory experiences, all minoritized groups still face discrimination due to systemic inequalities (Lei et al., 2022). As such, identifying shared experiences of oppression among Asian American children may help to promote the development of (and later, solidify) their critical racial consciousness in adulthood. Consequently, they may be more inclined and better equipped to engage in anti‐racism advocacy in solidarity with Black communities.

At the societal level, disruption of anti‐Blackness should be promoted through community‐based interventions and campaigns. For example, in response to Vietnamese/Asian‐Black interracial tension in Philadelphia, Pennsylvania, *Homeward Bound*, a political education youth organizing program for Vietnamese immigrant youth, was developed to empower youth to engage in critical reflection, leading them to question their anti‐Black biases and brainstorm solutions for change (Nguyen & Quinn, 2018). Among campaigns that are developed to disrupt anti‐Blackness, there is a need for national media campaigns that promote the increased representation of Black individuals in the media and challenge common stereotypes and biases associated with being Black. Additionally, the media can be a powerful tool for showcasing solidarity between Asian and Black individuals by highlighting stories of collaboration and shared struggles. By featuring Asian and Black communities working together in solidarity, the media can challenge divisive narratives and foster a sense of unity, empathy, and mutual support. This can help eliminate misconceptions and promote critical reflection among viewers, ultimately cultivating a heightened awareness and understanding of systemic inequities and racial inequalities and injustices.

## CONCLUSION

In the context of the pervasive model minority myth and anti‐Blackness within the Asian American community, this study sought to examine the role of parental socialization in shaping attitudes and behaviors toward the Black community. The findings demonstrated that parents' anti‐Black messaging was related to greater fear of and less empathy toward Black individuals, which ultimately predicted a lower perceived ability to engage in anti‐racism advocacy among Asian American emerging adults. As these emotions (fear, empathy) likely reflect their perspective on anti‐Blackness and Black–Asian relations, studies and interventions are needed to address and negate beliefs, attitudes, and messaging that perpetuate the racial triangulation model, wherein Asian Americans are valorized at the expense of the Black community. By facilitating the development of critical consciousness at the individual and systemic level, conversations regarding anti‐Blackness can evolve toward advocacy for the Black community and collective resistance against systemic oppression faced by people of color.

## Data Availability

Data used in this study may be available upon request.
